# Age and prior blood feeding of *Anopheles gambiae* influences their susceptibility and gene expression patterns to ivermectin-containing blood meals

**DOI:** 10.1186/s12864-015-2029-8

**Published:** 2015-10-15

**Authors:** Jonathan A. Seaman, Haoues Alout, Jacob I. Meyers, Mark D. Stenglein, Roch K. Dabiré, Saul Lozano-Fuentes, Timothy A. Burton, Wojtek S. Kuklinski, William C. Black, Brian D. Foy

**Affiliations:** Department of Microbiology, Immunology and Pathology, Arthropod-borne and Infectious Diseases Laboratory, Colorado State University 1692 Campus Delivery, Fort Collins, CO 80525 USA; Institute de Recherche en Sciences de la Santé (IRSS)/Centre Muraz, Direction Régionale de l’Ouest, 399 Ave de la Liberté, Bobo Dioulasso, Houet 10400-000 Burkina Faso

**Keywords:** *Anopheles gambiae*, Ivermectin, Vector control, Malaria transmission, RNA-seq

## Abstract

**Background:**

Ivermectin has been proposed as a novel malaria transmission control tool based on its insecticidal properties and unique route of acquisition through human blood. To maximize ivermectin’s effect and identify potential resistance/tolerance mechanisms, it is important to understand its effect on mosquito physiology and potential to shift mosquito population age-structure. We therefore investigated ivermectin susceptibility and gene expression changes in several age groups of female *Anopheles gambiae *mosquitoes.

**Methods:**

The effect of aging on ivermectin susceptibility was analyzed in three age groups (2, 6, and 14-days) of colonized female *Anopheles gambiae* mosquitoes using standard survivorship assays. Gene expression patterns were then analyzed by transcriptome sequencing on an Illumina HiSeq 2500 platform. RT-qPCR was used to validate transcriptional changes and also to examine expression in a different, colonized strain and in wild mosquitoes, both of which blood fed naturally on an ivermectin-treated person.

**Results:**

Mosquitoes of different ages and blood meal history died at different frequencies after ingesting ivermectin. Mortality was lowest in 2-day old mosquitoes exposed on their first blood meal and highest in 6-day old mosquitoes exposed on their second blood meal. Twenty-four hours following ivermectin ingestion, 101 and 187 genes were differentially-expressed relative to control blood-fed, in 2 and 6-day groups, respectively. Transcription patterns of select genes were similar in membrane-fed, colonized, and naturally-fed wild vectors. Transcripts from several unexpected functional classes were highly up-regulated, including Niemann-Pick Type C (NPC) genes, peritrophic matrix-associated genes, and immune-response genes, and these exhibited different transcription patterns between age groups, which may explain the observed susceptibility differences. Niemann-Pick Type 2 genes were the most highly up-regulated transcripts after ivermectin ingestion (up to 160 fold) and comparing phylogeny to transcriptional patterns revealed that NPCs have rapidly evolved and separate members respond to either blood meals or to ivermectin.

**Conclusion:**

We present evidence of increased ivermectin susceptibility in older *An. gambiae* mosquitoes that had previously bloodfed. Differential expression analysis suggests complex midgut interactions resulting from ivermectin ingestion that likely involve blood meal digestion physiological responses, midgut microflora, and innate immune responses. Thus, the transcription of certain gene families is consistently affected by ivermectin ingestion, and may provide important clues to ivermectin’s broad effects on malaria vectors. These findings contribute to the growing understanding of ivermectin’s potential as a transmission control tool.

**Electronic supplementary material:**

The online version of this article (doi:10.1186/s12864-015-2029-8) contains supplementary material, which is available to authorized users.

## Background

*Anopheles gambiae* is one of the most significant malaria vectors in sub-Saharan Africa. Current vector control methods predominantly rely on insecticide-based tools, including indoor residual spraying (IRS), and long-lasting insecticide-treated bed-nets (LLINs) [1]. The four classes of vector control insecticides: pyrethroids, organophosphates, organochlorines, and carbamates, have all been co-opted from agricultural use. So while highly successful, the combination of widespread agriculture and vector control insecticide applications has fostered genetic and behavioral resistance among *An. gambiae* and other malaria vector species [2]. Insecticide resistance is threatening the recent gains in controlling malaria worldwide, especially in Africa, highlighting the need for novel anti-vector strategies and application methods.

Ivermectin (IVM) is a semi-synthetic member of the avermectin drug family, a series of 16-membered lactone endectocides first isolated from *Streptomyces avermetilis* in 1975 [3]. It is commonly used in humans, livestock and pets to control endoparasites, including parasitic helminths such as *Onchocerca volvulus* [4, 5], *Haemonchus contortus* [6] and *Dirofilaria immitus* [7], and ectoparasites including scabies mites [8], bot flies and ticks [9, 10]. Due to IVM’s strong safety profile in humans [11] and the fact that adverse reactions are rare and generally mild [12, 13], it is distributed to entire communities in mass drug administrations (MDA) once or twice per year for control of onchocerciasis and lymphatic filariasis in Africa. Following from its activity against ectoparasite arthropods, IVM has been studied for its ability to cause mortality in multiple arthropod disease vectors that feed upon, but do not infest, their hosts such as tsetse flies, sandflies and mosquitoes [14–16]. IVM is particularly potent against Anopheline mosquitoes, including *An. gambiae*, causing significant mortality, delayed re-feeding, and reduced fertility when ingested via a blood meal [17–23]. MDA of IVM in West African villages was shown to significantly reduce *An. gambiae* survivorship and sporozoite rates in field-captured mosquitoes [22, 24, 25]. Clinical trials with repeated IVM MDA are underway to examine the potential to achieve sustained malaria control in communities.

As an anti-malaria vector tool, IVM has a unique administration route in that it is ingested through a blood meal rather than physical contact. In addition, IVM has a novel mode of action (MOA) for malaria vectors by acting as an agonist of glutamate-gated chloride channels [26], causing flaccid paralysis and eventual death. Due to its short pharmacokinetic persistence, many vectors will ingest a sub-lethal dose if they bite people more than 2 days post treatment [21]. To maximize its effectiveness, it is important to understand the gene expression and other molecular events in the vector following IVM ingestion to further characterize the drug’s effect on mosquito physiology, and the potential for mosquito resistance. Similar studies examining mosquitoes after exposure to traditional insecticides have mainly identified transcriptional changes to cuticular proteins, transporters, mitochondrial respiratory mechanisms, and detoxification processes. [27, 28]. Transcriptional responses to avermectin exposure have been studied in *Pediculus humanus humanus* [29] and *Rhipicephalus microplus* [30]*,* with both studies observing elevated transcription of ATP-binding cassette (ABC) transporters. *Drosophila* have been selected for resistance against IVM and abamectin in two different studies, with target-site insensitivity attributed to IVM resistance [31] and increased expression of ABC transporters attributed to abamectin resistance [32].

Evaluation of a compound’s potential as a transmission control tool should involve characterization of the molecular events surrounding vector exposure. Specifically, we wanted to determine whether gene expression patterns in *An. gambiae* reflect up-regulation of classical insecticide detoxification genes in response to IVM or whether novel gene classes may be up or down regulated. Additionally, because of the relatively long extrinsic incubation period associated with malaria transmission and the lethal effect of IVM on vectors, it is important to understand IVM’s ability to affect mosquito population age structure and mosquito vectorial capacity. To this end, we first compared IVM susceptibility among different age groups of female *An. gambiae.* Next, RNA-seq technology was utilized to assess transcriptional responses following IVM ingestion within and between these age groups. Expression patterns were validated with quantitative PCR on individual samples and on naturally-exposed mosquitoes that blood fed on a treated person.

## Methods

### Mosquito colony

*An. gambiae* s.s (G3 strain) were reared at the Arthropod-Borne and Infectious Diseases Laboratory at Colorado State University (CSU). This colony was kept at a constant 28–29 °C and 70–80 % relative humidity on a 14:10 light:dark cycle. Larvae were hatched in room temperature water, provided ground TetraMin® fish food daily, and were allowed to pupate and emerge into an enclosed mesh-covered cage where they were provided water and 10 % sucrose *ad libitum*.

### Mosquito bioassay and feeding schedule

Three age groups of mosquitoes were reared and assayed for IVM-induced mortality; 2 days-post-emergence (DPE), 6DPE and 14DPE. Each group was split into treatment and control groups, receiving an IVM or control blood meal at the specified age. Feeding was done using defibrinated calf blood (Colorado Serum Company, Denver, CO) containing 11.75 ng/ml IVM (10 mg/ml stock solubilized in dimethyl sulfoxide (DMSO) and diluted in phosphate buffered saline (PBS)). This concentration was chosen based on the human pharmacokinetic curve for IVM, corresponding approximately to the concentration found in human blood 36 hours post ingestion and representing a sub-lethal dose (<LC_50_) to *An. gambiae* [21]*.* Control blood meals contained the same volume of DMSO alone in PBS. Blood feeding was performed using glass bell feeders (Lillie Glass Feeders, Smyrna, GA) with membranes of hog sausage casings, and held at 37 °C. Following feeding, mosquitoes were knocked down at 4 °C, sorted on chilled plates, and only fully engorged females were kept. For the 6DPE and 14DPE groups, mosquitoes were fed normal blood meals and allowed to oviposit as they aged to emulate natural conditions; only those that engorged on these normal blood meals were carried into later age classes to maintain intergroup similarities. Thus, for 2DPE groups, the IVM blood meal (or corresponding control) they ingested on day 2 was their first and only blood meal. 6DPE groups received one normal blood meal on day 2, allowed to oviposit on day 4, and then were given their IVM-containing blood meal (or corresponding control) on day 6. 14DPE groups received three normal blood meals on days 2, 6, and 10, allowed to oviposit 2 days after each, and then were given their IVM or control blood meal on day 14. Following treatment blood meals, mortality was recorded daily for five days. A total of five biological replicates were performed for each age group.

### Statistical analysis of bioassays

Survival analysis of bioassays was performed on pooled replicates for each age (Log-rank (Mantel-Cox) test) to test for significant mortality from IVM. Comparison of mortality between age groups was analyzed using the Mantel-Haenszel hazard ratio with 95 % confidence intervals to account for control mortality (Prism 5; GraphPad Software, Inc.).

### RNA-seq library preparation

Mosquito bioassay feeding schedules were repeated exactly as above using 2DPE and 6DPE mosquito groups fed on control or IVM-treated blood. Twenty-four hours after blood-feeding, 10 fully engorged females were flash-frozen and stored at −80 °C awaiting RNA extraction. Groups of 10 unfed 2DPE and 6DPE females were also collected and flash-frozen at the same time. Two biological replicates were performed at each age, resulting in 20 blood-fed individuals per treatment. RNA was isolated from each individual sample using a Trizol® (phenol-chloroform) extraction procedure and genomic DNA was removed using Turbo™ DNase (Ambion® Life Technologies, Grand Island, NY). Following isolation, RNA was analyzed with an Agilent 2100 Bioanalyzer to visually check for RNA degradation. Individual mosquito RNA was then quantified with a Qubit® 2.0 Fluorometer (Life Technologies, Grand Island, NY) and an equal amount of RNA (200 ng) from each individual was used to pool in groups of ten. Pooled RNA was then subjected to TruSeq Stranded mRNA processing (Illumina, Inc., San Diego, CA). This involved mRNA purification by poly-T oligo attached magnetic beads, fragmentation, cDNA synthesis, adapter ligation, and amplification, resulting in 12 barcoded cDNA libraries. Samples were sent to Beckman Coulter Genomics (Boston MA), where they were sequenced on an Illumina HiSeq 2500 to produce 338 million 2x100-bp paired-end reads. The total number of sequences obtained for each library can be found in Additional file 1: Table S1.

### Sequencing data analysis

Data analysis was performed on a Linux platform using GMAP and GSNAP [33, 34] for indexing and alignment, respectively, and Trimmomatic [35] for quality control and removal of singletons. Reads were aligned to the AgamP3.22 genome build (VectorBase, http://www.vectorbase.org, *Anopheles gambiae* PEST, AgamP3.22) [36]. Low expression tags were filtered using a threshold of 100 counts per million (CPM). Statistical analysis was performed using the edgeR package [37] for R software [38]. Counts were first fit to a negative binomial generalized log-linear model (glmFit procedure) and the dispersion was estimated by plotting the Biological Coefficient of Variation (BCV) for each biological replicate (Additional file 1: Figure S1). Differential expression (DE) analysis was then performed using the glmLRT procedure. Significant DE genes were identified using the exact test for two groups of negative-binomial counts with *α* = 0.05 and the Benjamini-Hochberg false discovery rate *p*-value correction [39]. Overall, three blood feeding states were analyzed; non-blood-fed (NBF), blood-fed (BF), and IVM-blood-fed (IVM). DE contrasts are denoted in a X:Y format, whereby X is up- or down-regulated compared to Y. For example, a positive fold-change (FC) of 2.5 in the 2IVM:2BF contrast would indicate 2.5 up-regulation in the 2DPE IVM-blood fed group relative to the 2DPE control blood fed group. Likewise, a negative value would indicate down-regulation.

### Gene ontology term associations

Gene Ontology (GO) terms for DE genes were retrieved using g:Profiler [40] with the “significant only” option un-checked so as to retrieve all GO terms. While GO terms fall into three different groups (biological process, molecular function, and cellular component), only biological process and molecular function were used for this analysis. Upon classifying GO terms into more general categories, the number of unique transcripts linked to each broad category was plotted for each contrast and direction of differential expression. Because a single gene may return multiple GO terms, transcripts were allowed to fall into multiple categories.

### Phylogenetic analysis

To construct a bootstrapped phylogenetic tree, SEQBOOT in PHYLIP 3.69 [41] generated 1000 bootstrap copies. These were then analyzed by PROTDIST in PHYLIP to derive 1000 Dayhoff PAM (Point Accepted Mutation) matrices using the DCMut model [42] which is a version of the original PAM model [43]. The PAM model scales probabilities of change from one particular amino acid to another in terms of % change between two amino acid sequences. NEIGHBOR in PHYLIP then derived 1000 neighbor-joining trees [44] with *An. christyi* treated as the outgroup. CONSENSE in PHYLIP then derived a consensus tree and reported the number of times that each branch was recovered in the 1000 trees. TreeGraph_2.0.47-206_beta [45] was used to draw the consensus tree.

### Real-time quantitative PCR confirmation

As an external validation of RNA-seq results, RT-qPCR primers were developed for the following five genes that showed significant differential expression following an IVM blood-meal: glutathione-S-transferase, delta class, member 3 (GSTd3, AGAP004382) ornithine decarboxylase (ODC, AGAP011806), ABC transporter, subfamily A, member 3 (ABCA3, AGAP006380), thioredoxin peroxidase (Tpx, AGAP011824), and cytochrome P450, family 9, subfamily J, member 5 (CYP9J5, AGAP012296). Validation was done only using 2DPE mosquitoes. Gene expression was normalized to ribosomal protein S7 (RPS7, AGAP010592) as an internal reference gene. Expression of this gene from RNA-seq was consistent (not DE) between IVM and BF groups, justifying its use as a reference. Sequences for all primer pairs can be found in Additional file 2: Table S2. Primers were designed using the web-based PrimerQuest tool [46]. Technical duplicates were performed for each sample-primer pair. Upon TurboDNase treatment, individual samples were pooled and normalized, using 4 ng of input RNA for the SensiFast™ SYBR & Fluorescein One-step kit (BioLine, London, UK) and the suggested 3-step cycling conditions. RNA samples for those labeled “G3” were derived from the same total RNA samples used for RNA-seq library preparation (*An. gambiae* G3 strain raised at CSU), representing two biological replicates, each consisting of three individual mosquito RNA extracts pooled together. To analyze these transcription patterns in different samples, gene expression was also assessed in mosquitoes originating from West Africa. *An. gambiae* s.s. (IRSS strain) were recently-colonized at the Institut de Recherche en Sciences de la Santé, in the city of Bobo Dioulasso, Burkina Faso. Additionally, wild *Anopheles gambiae* s.s. (fourth instar larvae and pupae) were captured from a rain water pool in the Kodeni district of Bobo Dioulasso, and reared into adults. Both the IRSS (colonized) and Kodeni (wild-type) mosquitoes were separated into treatment and control groups at 1DPE, and then at 2DPE the treatment groups were blood fed on the arm of a human who had taken a standard oral dose of IVM (150 μg/kg) 36-h prior, while the control group fed on a different, untreated human. This study was approved by the ethics committee of Colorado State University and written consent was obtained from each participating individual (IRB protocols 09-1148H and 12-3597H). PCR was performed on the Kodeni samples for species identification [47], differentiating between unique SNPs specific to *An. gambiae* and *An. arabiensis.* Using this method, only a total of four *An. gambiae* samples were identified, two from each treatment (IVM and control). Because of this limitation, qPCR results from these field samples represent pooled total RNA from two individuals as opposed to the three used for G3 and IRSS samples. Fold-changes in gene expression were calculated using the ΔΔCt method [48]. A Spearman rho correlation analysis (GraphPad Prism 6) was performed between the RNA-seq and RT-qPCR fold-change results using the same G3 RNA samples.

## Results and discussion

### Aging effects on IVM susceptibility

**Fig. 1:**
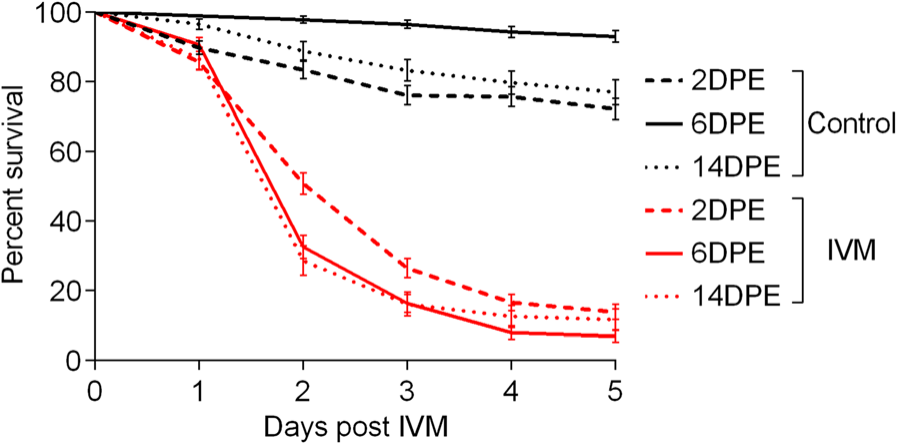
Effect of aging on mosquito survival following ingestion of 11.75 ng/mL IVM in calf blood. Mortality was recorded for five days following IVM-containing blood ingestion (and corresponding controls) in the three age groups analyzed. All IVM groups exhibited significant mortality compared with their controls (Log-rank Mantel-Cox test, *p* < 0.0001). Five biological replicates were performed at each age with the following total sample sizes: 2DPE (*n* = 258), 6DPE (*n* = 200), 14DPE (*n* = 112). Data presented as mean and S.E

**Fig. 2:**
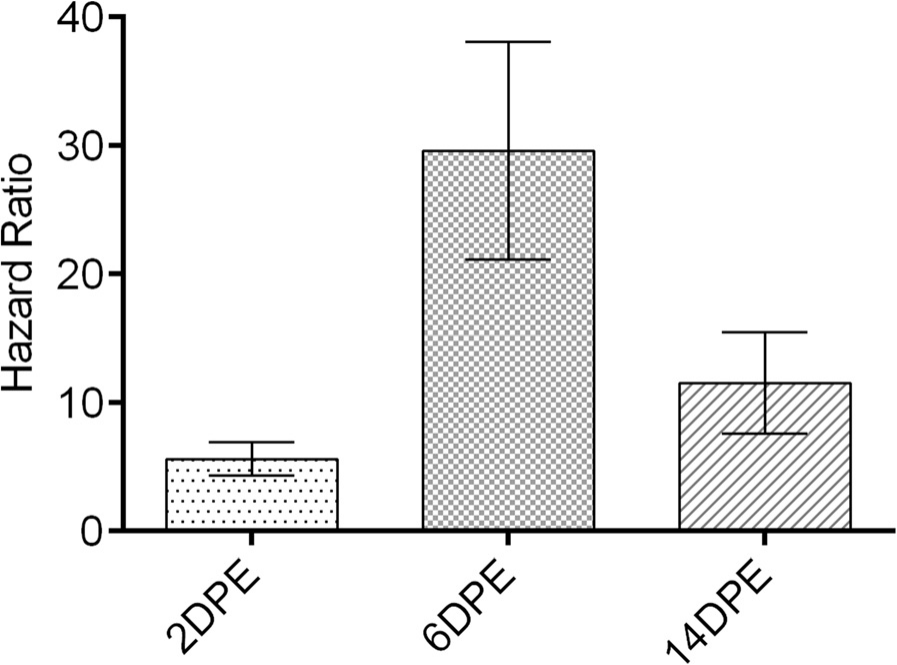
Mantel-Haenzel hazard ratio associated with IVM ingestion in three age groups. Mantel-Haenzel Hazard ratio computed by comparing each IVM treated group to its corresponding control, thus factoring in each age group’s control mortality. Error bars represent the 95 % confidence interval of the hazard ratio. Each age group showed significantly different hazard ratios as there was no crossover between 95 % CIs. 2DPE: *HR* = 5.47, 95 % *CI* = 4.20 – 7.14; 6DPE: *HR* = 27.89, 95 % *CI* = 19.84 – 39.02; 14DPE: *HR* = 11.49, 95 % *CI* = 7.54 – 17.52

Significant mortality (*p* < 0.0001) was observed in the IVM-treated mosquitoes in each age group (Fig. 1). When comparing mortality in control blood fed mosquitoes between age groups, 6DPE mosquitoes had significantly higher survival than the 2DPE control group, likely reflecting the substantial cost of ingesting the first blood meal in life. They also survived significantly better than the 14DPE group, likely reflecting senescence caused by the accumulated effects of serial blood meals and ovipositions [49]. To most accurately compare the effect of IVM between age groups, the Mantel-Haenszel hazard ratio (HR) was calculated to assess the odds of death in treatment groups relative to their paired controls (Fig. 2). Comparing the 95 % confidence intervals for these HRs, mortality induced by this discriminating IVM concentration was lowest in 2DPE (*HR* = 5.47; 95 % CI: 4.20 – 7.14, *N* = 258), highest in 6DPE mosquitoes (*HR* = 27.89; 95 % CI: 19.84 – 39.02, *N* = 200), while the mortality was intermediate in 14DPE mosquitoes (*HR* = 11.49; 95 % CI: 7.54 – 17.52, *N* = 112).

Several studies have investigated the effect of mosquito aging on insecticide susceptibility and/or resistance profile maintenance. In the majority of studies, insecticide susceptibility increases with aging [50–53]. In a study with *An. gambiae,* Rajatileka et al. [50], demonstrated that blood feeding status had little to no impact on DDT, bendiocarb, and deltamethrin susceptibility, but that age was the more dominant factor contributing to insecticide susceptibility. In contrast, two *Culex* and *Anopheles* studies revealed increased insecticide tolerance after either single or multiple blood meals, as well as maintenance of resistant profiles [54, 55]. Another study investigating the effect of bloodfeeding on permethtrin tolerance in both a susceptible and resistant strain of *An. funestus* revealed that a previous blood meal had no effect on tolerance in the susceptible strain but significantly increased tolerance in the resistant strain [56]. In our study, we only tested a strain of *An. gambiae* that is susceptible to standard insecticides as well as to IVM, as we are not aware of any strains that display IVM resistance. Our data reveal that a previous blood meal and/or aging in *An. gambiae* significantly decreases tolerance to IVM in a susceptible strain, somewhat contrary to studies using classical insecticides.

Exposure to IVM is intrinsically linked to simultaneous blood meal ingestion and digestion. Our results uniquely show that rather than a steady increase in susceptibility with aging, it is highest in middle-aged mosquitoes that have already processed an earlier blood meal. We previously examined the IVM susceptibility between 2DPE and 8DPE *An. gambiae*, both ingesting their first blood meal in life. [21]. In contrast to our current data, those data identified no differences in susceptibility to sub-lethal IVM concentrations between age groups, suggesting that ingestion of a prior blood meal is the primary cause of the differential IVM susceptibility between young and old mosquitoes. Ingestion and processing of a blood meal is a taxing physiological commitment as the mosquito ingests up to three times its weight in blood within seconds to minutes [57]. Digesting this meal necessitates rapid diuresis as well as osmotic balancing, and it generates reactive oxygen species and the liberation of cytotoxic heme molecules [58]. The interaction of IVM with these and other blood meal associated molecular and physiological events is undoubtedly complex and not understood. The drug is known to be lipophilic and incorporates into cell membranes [59, 60], but its primary target, the glutamate-gated chloride channel, was shown to be present in *An.gambiae* nervous tissues of the thorax and head but absent from abdomen [26]. Given this lack of understanding, we analyzed differential gene expression in control and IVM-treated mosquitoes from the 2DPE and 6DPE groups.

### RNA-seq – differential expression analysis

RNA-seq was performed on two biological replicates of 10 individuals from both the 2DPE and 6DPE groups, chosen because they exhibited the greatest susceptibility difference to a discriminating IVM concentration. Sequencing, alignment, and annotation identified 13,465 unique transcripts. Filtering of low-expression tags and normalization reduced this number to 2,307 which were then subjected to differential expression (DE) analysis. The complete list of DE genes in each contrast can be found in Additional files 3, 4, and 5.

**Fig. 3:**
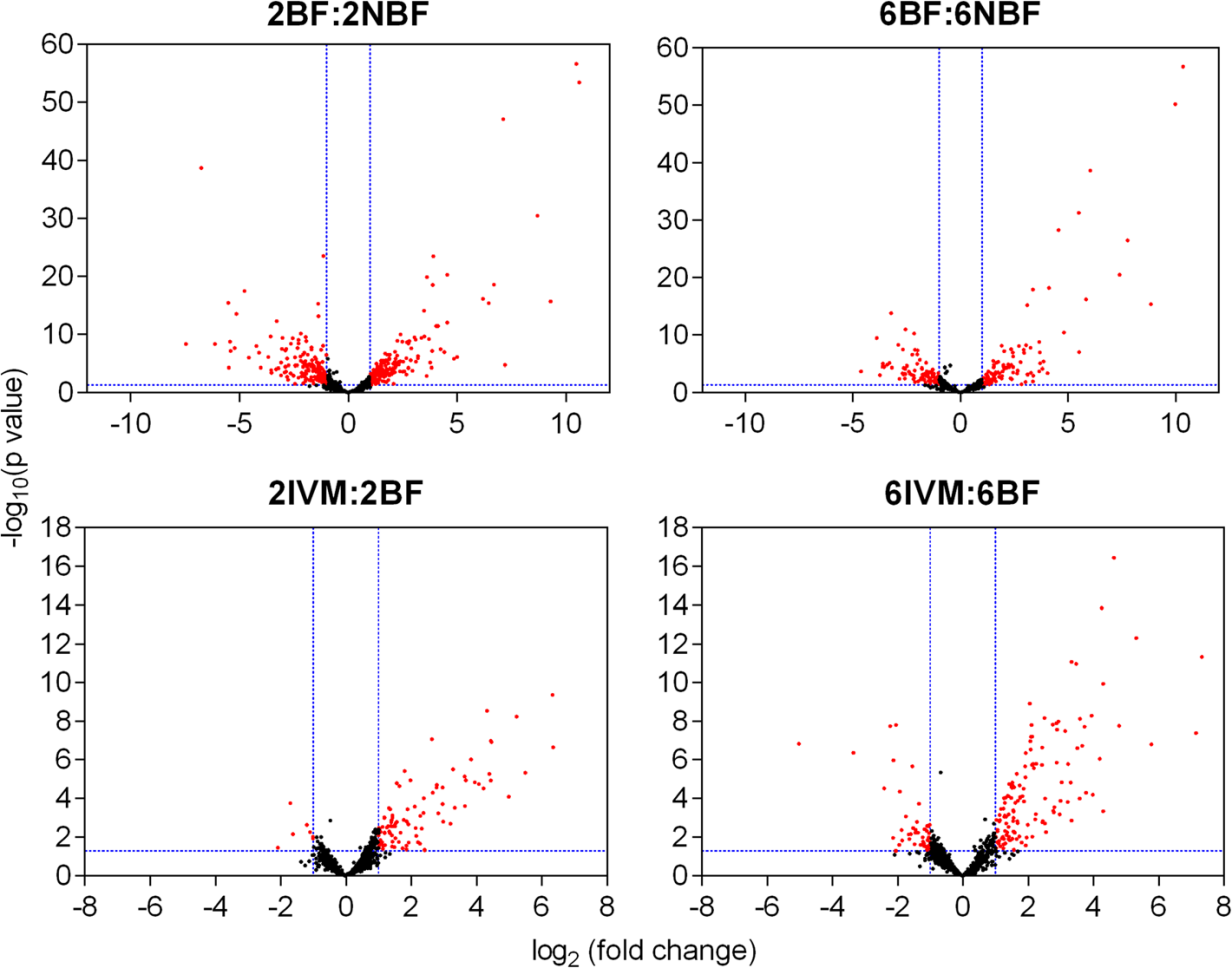
Visualization of differential expression using volcano plots. Differential gene expression depicted as volcano plots for the BF:NBF and IVM:BF contrasts at both age points. Horizontal blue dotted lines indicate significance threshold (*p* < 0.05) whereas vertical lines indicate fold-change threshold (≥2-fold). Red points represent significantly differentially expressed transcripts by these criteria. Positive x-values represent up-regulation and negative *x*-values represent down-regulation

**Fig. 4:**
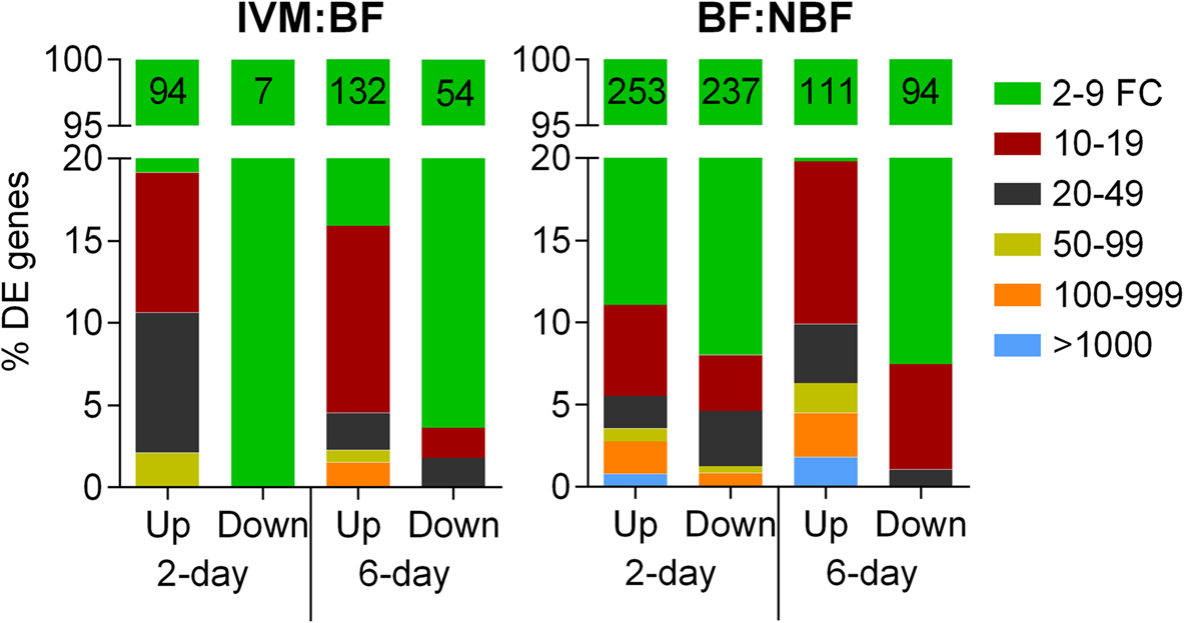
Distribution of fold-changes and total number of DE genes associated with each contrast. Fold-changes were separated into 6 ranges (2–9, 10–19, 20–49, 50–99, 100–999, and >1000). The percentage of transcripts in each range was plotted for each contrast, separated by fold-change direction. The total number of DE transcripts in each contrast and direction is indicated numerically at the top of each bar

Figures 3 and 4 summarize the results of DE analyses in the BF:NBF and IVM:BF contrasts. Transcripts were considered significantly differentially expressed if *p* < 0.05 and fold-change ≥ 2, indicated by red points on Fig. 3. Control blood feeding of both age groups (Figs. 3 and 4; BF:NBF) generally resulted in symmetrical changes, with similar numbers of up- and down-regulated transcripts. Consistent with previous microarray and sequencing studies [61–63], blood feeding alone caused dramatic DE, with blood digestion and vitellogenesis genes up-regulated up to 1000-fold and down-regulated transcripts more modestly changed (Figs. 3 and 4). One limitation of our study is that for technical and cost considerations, we only performed RNA-seq on two biological replicates for each age group, and we only analyzed a single time point post-blood meal digestion (24 h). Having three replicates may have made our analysis more precise, and it is clear that DE changes fluctuate significantly from the time of blood meal ingestion to completion of digestion [63, 64].

Considering age, it is notable that control blood feeding (BF:NBF) by 2DPE mosquitoes resulted in more than double the amount of DE transcripts than 6DPE mosquitoes (253 vs 111 up-regulated and 237 vs 94 down-regulated; Figs. 3 and 4). This is likely due to the fact that the 2DPE mosquitoes were digesting their first blood meal, while the 6DPE mosquitoes were digesting their second blood meal. Indeed, most *An. gambiae* have a ‘pre-gravid’ stage following emergence [65], whereby they utilize their first blood meal to replenish energy reserves and the second to complete vitellogenesis and mature their first egg batch. It may be that the significantly greater mortality in control mosquitoes that we observed in 2DPE mosquitoes relative to 6DPE mosquitoes (Fig. 1) is associated with this pre-gravid state and/or the higher number of DE genes in this group.

Analysis of DE between the 6DPE and 2DPE age groups within the same treatment categories (6IVM:2IVM, 6BF:2BF, and 6NBF:2NBF) (Additional file 6: Figure S2, Additional file 5) revealed few significantly DE genes, with most appearing in the NBF contrast. However, because these contrasts do not factor in the corresponding control groups, these analyses are less informative in studying the transcriptional response to IVM, and will not be discussed further. An alternative method to analyze the transcriptional response to IVM is to first compare the age-specific BF:NBF contrasts with the IVM:NBF contrasts, subtracting out the blood-feeding effect by focusing only on those DE genes unique to the IVM:NBF contrast. As expected, many DE genes are common between the IVM:BF and subtracted IVM:NBF contrasts within each age group (Additional files 7 and 8: Tables S6.4 and 7.4) (*n* = 22 in 2DPE, *n* = 59 in 6DPE) and have very similar fold-change values in both contrasts. However, there remained numerous DE genes unique to the subtracted IVM:NBF contrasts (Additional files 7 and 8: Tables S6.6 and 7.6) that may represent a synergistic interaction between the blood feeding and ivermectin effect. The fold-change values of the genes tended to be modest (−4 < FC < 4) and an examination of their gene descriptions does not identify any particular biological process.

**Fig. 5:**
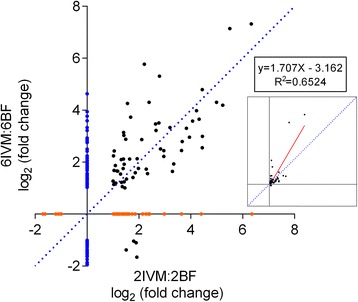
Linear regression analysis comparing the gene expression after IVM ingestion between age groups. 62 transcripts were common to both contrasts while an additional 124 transcripts were unique to the 6-day contrast and 39 were unique to the 2-day contrast. Four transcripts showed a different direction of DE between the two contrasts while the remaining 58 showed the same direction of DE. The larger plot shows log_2_ transformed fold-change values while the smaller plot shows untransformed data and the accompanying linear regression line and equation

In contrast to control blood feeding, IVM-containing blood meals resulted in asymmetrical transcriptional changes with the majority of significantly DE transcripts showing up-regulation at both ages (Figs. 3 and 4). Additionally, more genes were differentially-regulated in response to IVM in the 6DPE group relative to the 2DPE group, and the fold-changes were overall higher. Figure 5 shows a linear regression analysis comparing those significantly DE transcripts common to the IVM response in both 2-day and 6-day groups, while transcripts unique to one contrast are shown on their respective axes. This again revealed a trend towards higher up-regulation in 6DPE mosquitoes compared with 2DPE mosquitoes (linear regression, inset graph). In addition, four DE transcripts were down-regulated in 6DPE mosquitoes but up-regulated in 2DPE mosquitoes, as seen by their presence below the X-axis. The protein products of these transcripts are ATP-dependent RNA helicase DDX5/DBP2, tyrosine-3-monooxygenase, rap55, and maternal protein exuperantia. It is possible that one or more of these four transcripts are associated with the differential IVM susceptibility of 6DPE vs. 2DPE mosquitoes, but this is not obvious from their descriptions and all have only modest fold-change within each age group.

**Fig. 6:**
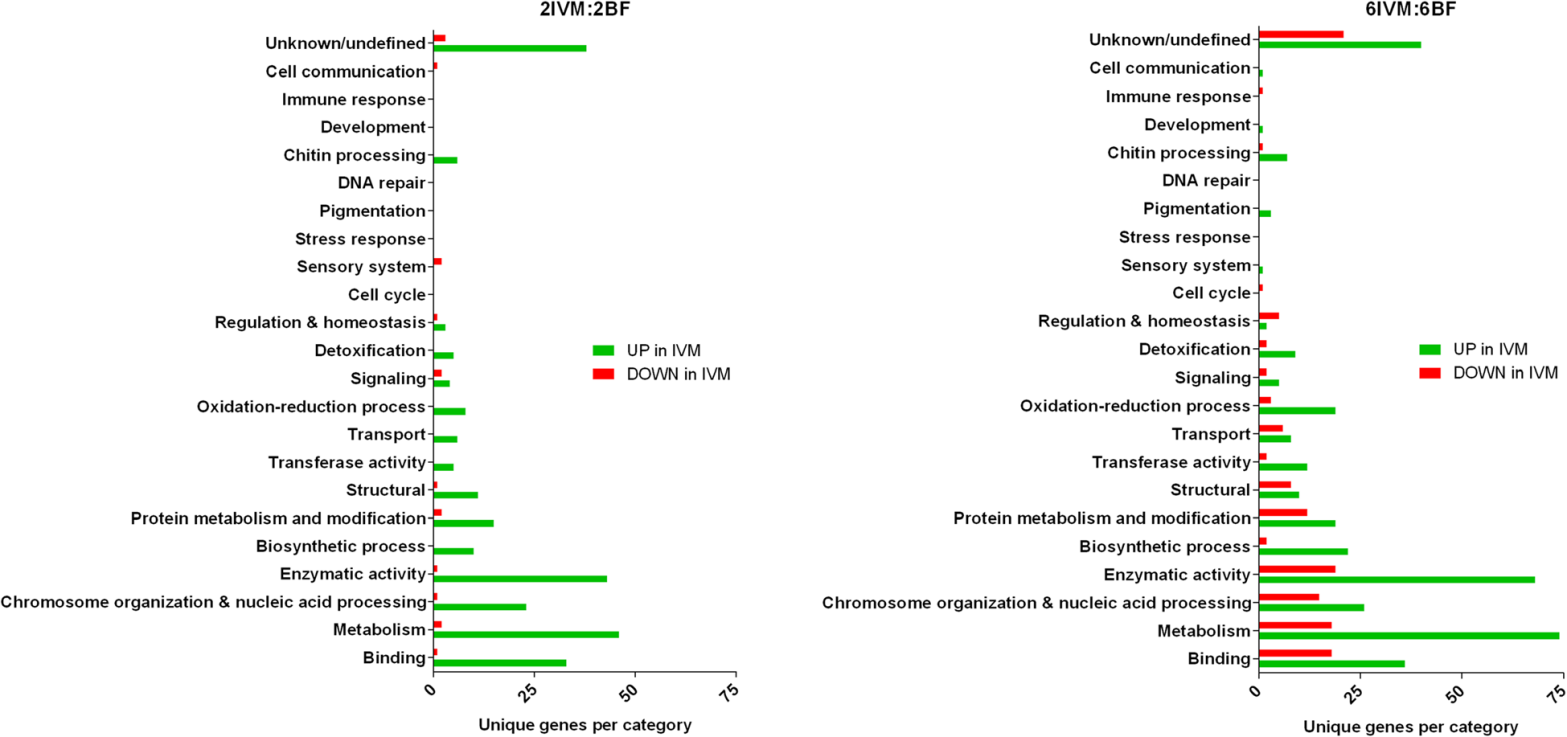
Gene Ontology terms associated with DE genes following IVM ingestion. Gene ontology terms associated with differentially expressed genes 24 h following an IVM blood feed in both 2 and 6-day old mosquitoes. GO terms were gathered with g:Profiler and manually sorted into the 23 functional categories. Only biological process and molecular function terms were used, cellular component terms were removed. Individual transcripts were allowed to fall into multiple categories, based on the GO terms returned, to more accurately reflect the multi-functional aspect of many enzymes

Gene ontology annotations of DE genes in the IVM:BF contrasts in both age groups showed a similar category distribution pattern (Fig. 6), with the majority of transcripts related to: binding, metabolism, chromosome organization & nucleic acid processing, and enzymatic activity. Additionally, a significant number of DE genes were described as ‘Unknown/undefined’, which is attributed to the incomplete annotation of the *An. gambiae* genome. Lastly, specific to the 6IVM:6BF contrast, several ontology categories had a high-proportion of down regulated genes relative to up-regulated genes (cell cycle, regulation & homeostasis, transport, structural).

### RT-qPCR validation

**Fig. 7:**
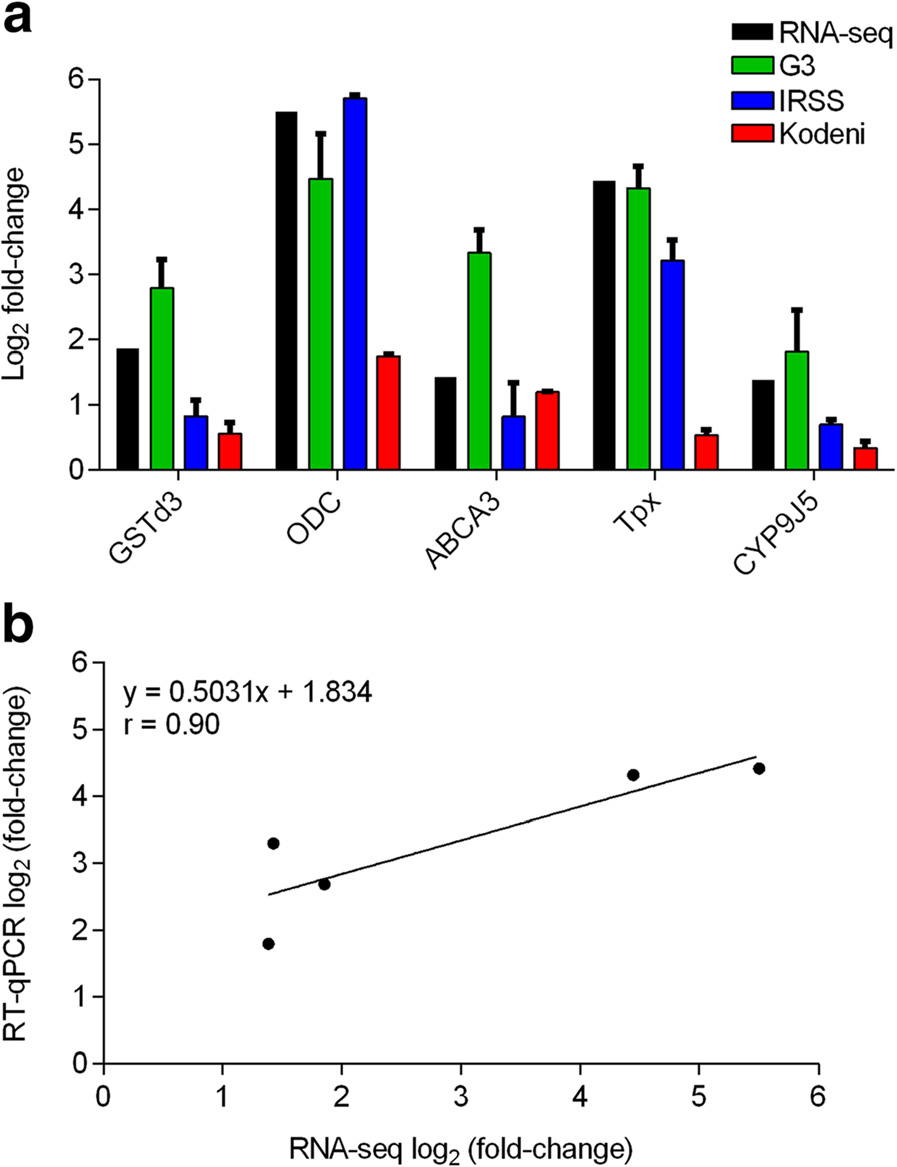
RT-qPCR validation of RNA-seq results. Five genes were selected for DE confirmation in the same RNA samples used for RNA-seq (G3), a laboratory raised colony from west-Africa (IRSS), and wild-caught mosquitoes from the Kodeni region of Burkina Faso (Kodeni). A: Log_2_ fold-change comparison with standard deviation (does not apply to RNA-seq results), B: Correlation analysis between RNA-seq and RT-qPCR log_2_ (fold-change) results from the same RNA samples (*An. gambiae* G3 strain). Spearman correlation coefficient is displayed (r). GSTd3: glutathione-S-transferase, delta class, member 3; ODC: ornithine decarboxylase; ABCA3: ABC transporter, subfamily A, member 3; Tpx: thioredoxin peroxidase; CYP9J5: cytochrome P450, family 9, subfamily J, member 5

We validated RNA-seq results of five DE transcripts by RT-qPCR (Fig. 7a). Expression values (log_2_ FC) from RNA-seq were compared to those derived from RT-qPCR analysis on the same RNA-samples (G3) and from RNA derived from both colony (IRSS) and wild (Kodeni) mosquitoes naturally-exposed in Burkina Faso. As expected, transcript-specific fold-change in the same G3 strain RNA samples was highly concordant between the RNA-seq and RT-qPCR methods, which was corroborated in the correlation analysis (Fig. 7b). Fold-change values in the naturally-exposed mosquitoes from Burkina Faso (IRSS and Kodeni) appear to generally agree with RNA-seq, however the degree of up-regulation was generally less and lowest in the wild-type (Kodeni) mosquitoes. The discrepancy could partially be explained by the fact that we were unable to determine the concentration of IVM acquired from the blood of the human volunteer (the concentration range is inferred from published pharmacokinetic curves following a standard oral dose), so the concentration was likely slightly different from that fed to the G3 strain via membrane feeders.

### Genes of interest

The following four groups of DE genes are highlighted based on their putative biological function and degree of differential expression that may relate to the observed age effect on IVM susceptibility, as well as increase our understanding of the broad physiological responses to IVM ingestion.

### Detoxification genes

Several DE transcripts (Table 1) were identified for their involvement with either detoxification of insecticides or oxidative-stress response. These include enzymes classically related to insecticide resistance such as glutathione-S-transferase (GST), cytochrome P450s, and ABC transporters. Half of the transcripts were differentially expressed at similar fold-changes in both age contrasts. Two oxidative-stress response genes were significantly DE only in the 6DPE contrast, but thioredoxin peroxidase was highly up-regulated in both age contrasts. While both age contrasts showed up-regulation of a cytochrome P450 upon IVM ingestion, the exact CYP gene differed (CYP9J5 in 2DPE, CYP6Z1 in 6DPE).

**Table 1:** Detoxification-related transcripts differentially expressed after IVM ingestion

Gene ID	Description	Function	2IVM:2BF FC	6IVM:6BF FC
AGAP011824	Thioredoxin peroxidase	Reduction of H_2_O_2_	21.77	27.61
AGAP004382	Glutathione-S-transferase, delta 3	Conjugation of GSH to xenobiotics	3.62	-
AGAP006380	ABC transporter, subfamily A, member 3	Efflux pump	2.69	2.36
AGAP012296	Cytochrome P450 9 J5	Oxidation of toxic compounds	2.61	-
AGAP009463	ABC transporter, subfamily G, member 1	Efflux pump	-	−2.94
AGAP008219	Cytochrome P450 6Z1	Oxidation of toxic compounds	-	2.92
AGAP009738	Glutaredoxin	Redox homeostasis	-	2.39

Detoxification and oxidative-stress response related transcripts significantly differentially expressed upon IVM ingestion in both age groups

GSTs have been linked with resistance to all major insecticide classes, functioning to conjugate reduced glutathione to a target molecule to both neutralize electrophilic sites and render it more water soluble and easily excretable [66–69]. Cytochrome P450-mediated oxidation of insecticides has been widely studied as it relates to resistance [70–73], and increased ABC-transporter expression and activity in insects has been linked to detoxification of carbamates [74], organochlorines [75], organophosphates [76], and pyrethroids [77]. Similarly, ABC transporters have been studied for their potential involvement in the detoxification of IVM in *Pediculus humanus* [29], *Culex pipiens* [78], *Rhipicephalus (Boophilus) microplus* [30, 76], *Caenorhabditis elegans* [79], and *Chironomus riparius* [80]. The degree of up-regulation of the ABCA3 (AGAP006380) was largely the same between age groups and therefore would not explain the susceptibility differences observed. ABCG1, on the other hand, was down-regulated in the 6DPE group but not DE in the 2DPE comparison. Additionally, GSTd3 was up-regulated 3.6-fold in 2DPE but not DE in 6DPE. Taken together, there may be involvement of GSTs and ABC transporters in the response to IVM, however because fold-changes were generally mild, other factors are likely involved in explaining the differential age susceptibility.

### Niemann-pick type C genes

The Niemann-Pick type C-2 (NPC2) family of genes are of interest because members were among the most highly up-regulated transcripts in our experiments (Table 2). One member (AGAP002851) is among the most highly DE genes in response to blood feeding within 2DPE mosquitoes (148 fold in our data set), it remains highly up-regulated in 6DPE mosquitoes in response to regular blood feeding (10 fold), and it is the highest up-regulated transcript related to aging and termination of the pre-gravid state (55 fold, 6NBF:2NBF, Table 1). Other members of this gene family responded more modestly to blood feeding in both age groups. Two NPC2 genes (AGAP002847, AGAP002848) were highly up-regulated in response to IVM ingestion at both ages, but the transcription of AGAP002847 in particular almost doubled in 6DPE vs. 2DPE mosquitoes.

**Table 2:** Niemann-Pick family members differentially expressed in multiple contrasts

Gene ID	Name	2IVM:2BF FC	6IVM:6BF FC	2BF:2NBF FC	6BF:6NBF FC	6NBF:2NB FC
AGAP002851	NPC2	-	-	148.1	10.3	55.4
AGAP002847	NPC2	80.6	159.5	-	-	-
AGAP002848	NPC2	16.8	11.9	−11.7	-	-
AGAP012352	NPC2	-	-	3.6	6.3	-
AGAP028108	NPC2	-	-	−2.1	-	−2.2
AGAP013106	NPC2	-	-	−3.7	-	-
AGAP008137	NPC1	-	−2.0	-	-	-

Niemann-Pick transcripts significantly differentially expressed in response to blood feeding (BF:NBF), IVM ingestion (IVM:BF), and aging (6:2)

In humans, these proteins are involved in cholesterol trafficking and homeostasis in the endosomal/lysosomal system [81, 82], and defects in either of the type C proteins, NPC1 or NPC2, cause two hereditary childhood neurodegenerative diseases (Niemann-Pick C diseases type 1 and type 2, respectively) [83]. While NPC1 is a large transmembrane-associated protein found in late endosomes [82], NPC2 is a small secretion signal-containing protein that is both secreted and found in lysomsomes [82, 84]. NPC2 contains a conserved domain called ML (MD-2 [myeloid differentiation factor-2]-related Lipid-recognition) that binds cholesterol and other lipids, such as fatty acids [85]. This ML domain is also a key domain in the human proteins MD-1 and MD-2 (myeloid differentiation factors), where it binds bacterial lipopolysaccharide (LPS) and mediates cellular innate immune responses to LPS through co-interactions with toll-like receptor 4 [86–88].

In insects, NPC2-related proteins appear to carry out a variety of functions related to their lipid-binding domains, including lipid adsorption, lipid trafficking and metabolism, pathogen recognition and immune-regulation. In insects they have been named either MD2-like, NPC2-like, or simply ML, but to remain consistent with their annotations in VectorBase [36], we refer to them here as NPC2 proteins and also reference some of their previous ML designations. These proteins have been described in the dipteran species *Drosophila*, *Aedes*, *Culex,* and *Anopheles*. In *Drosophila*, the eight NPC2 genes are transcribed variably in time and space within embryos and at least two of the genes (NPC2a and NPC2b) are necessary for sterol homeostasis and ecdysteroid biosynthesis [89]. Furthermore, three members of the *Drosophila* NPC2 protein family (NPC2a, NPC2e, and NPC2h) have been shown to bind LPS, lipid A, peptidoglycan and lipoteichoic acid, and play a role in immune signaling pathways involved in antimicrobial peptide production [90]. In *Ae. aegypti*, NPC1 may facilitate dengue virus entry while NPC2 proteins may play a role in modulating mosquito immunity to dengue virus [91]. The *Anopheles* NPC2 proteins (ML proteins) were extensively highlighted in Dong et al. [92] where the thirteen members were designated AgMDL1-13. They found that AGAP012352 (AgMDL1) and AGAP002857 (AgMDL2) were significantly transcribed in *An. gambiae* midguts upon *P. falciparum* ookinete invasion and that RNAi silencing of the former agonized oocyst development and growth of both gram negative and gram positive bacteria upon challenge.

**Fig. 8:**
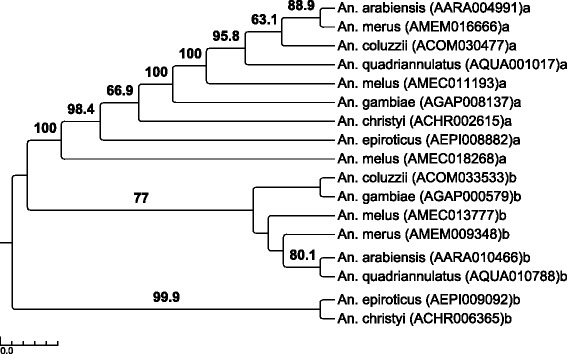
Phylogenetic tree of the NPC1 gene family in *Anopheles* species. The *Aedes aegypti* Niemann-Pick C1a protein (AAEL003325) was used to identify the orthologues in *Anopheles gambiae* s.l., *An. gambiae* s.s., *An. arabiensis*, *An. melas* (note the duplication), *An. merus*, *An. coluzzii*, and *An. quadrimaculatus*. The *Ae. aegypti* Niemann-Pick C1b protein (AAEL009531) was used to identify the orthologues in *Anopheles gambiae* s.l. For both NPC1a and NPC1b, *Anopheles christyi* and *An. epiroticus* were included as outgroups. The amino acid alignment contained 1818 characters. Branches with >50 % boostrap scores are labelled

**Fig. 9:**
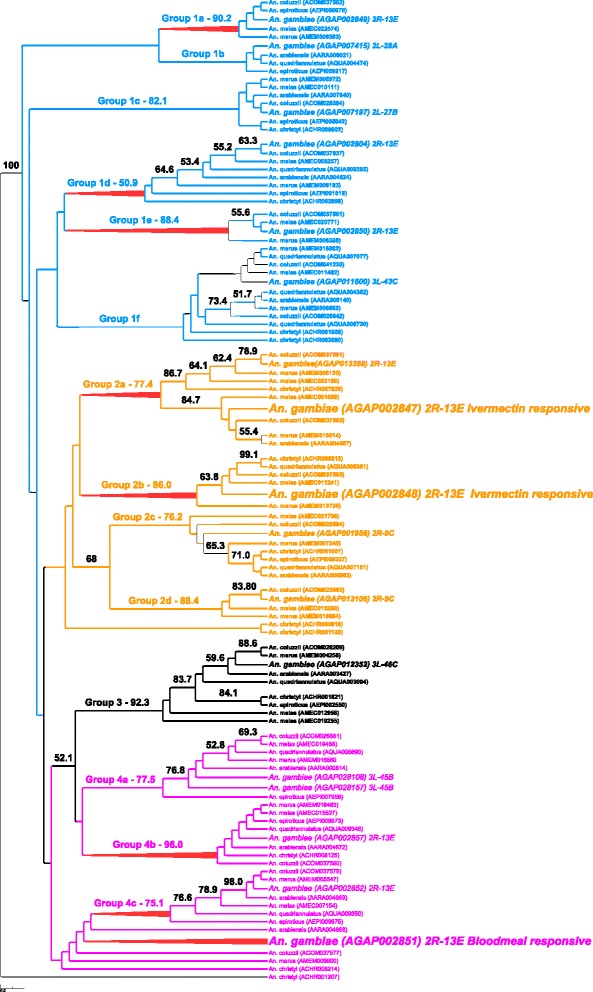
Phylogenetic tree of the NPC2 gene family in *Anopheles* species. *Aedes aegypti* Niemann-Pick C2 proteins from four phylogentic groups (AAEL009557, AAEL001650, AAEL004120, AAEL006854) were used in BLASTp to identify the orthologues in *Anopheles gambiae* s.l. *Anopheles christyi* and *An. epiroticus* were included as outgroups. BLASTp returned a total of 108 orthologues in *Anopheles* species, separated into four groups based on the *Ae. Aegypti* orthologue. BLASTp and phylogenetic analysis of AAEL009557 returned forty proteins in six subgroups (group 1, blue font), AAEL001650 returned thirty proteins in five subgroups (group 2, gold font), AAEL004120 returned nine proteins in one subgroup (group 3, black font), and AAEL006854 returned twenty-nine proteins in four subgroups (group 4, pink font) The genome location of *An. gambiae s.s* orthologues are listed after each gene. Groups with >50 % bootstrap support are red. *An. gambiae* orthologues appear in a larger font with the IVM and blood meal-responsive transcripts in the largest font. Note the duplication appearing in the same physical location in group 4a

We performed a phylogenetic analysis of NPC1 (Fig. 8) and NPC2 (Fig. 9) using current *An. gambiae s. l.* complex genome data from VectorBase [36] to try to better understand this diverse gene family and its myriad of functions. *Anopheles christyi* and *An. epiroticus* were included as outgroups in these analyses. As seen previously in Jupatanakul et al. [91], there are two distinct NPC1 proteins (NPC1a and NPC1b). Both NPC1a and NPC1b are monophyletic and have evolved during speciation within *An. gambiae* s.l., suggesting conserved functions, most likely related to basic sterol homeostasis and 20-hydroecdysone biosynthesis functions that have been ascribed to these proteins in *D. melanogaster* [89, 93, 94]. In our dataset, only *An. gambiae* NPC1a (AGAP008137) is modestly down-regulated and only in the 6IVM:6BF contrast (Table 2).

The NPC2 proteins from *An. gambiae* s.s [92], *D. melanogaster* [90] and *Ae. aegypti* [91] have been previously subjected to phylogenetic analysis. These latter two analyses placed these proteins in 4 broad groups. From this basis, BLASTp against *Ae. aegypti* representatives of the 4 previously-described groups (AAEL009557: group 1, AAEL001650: group 2, AAEL004120: group 3, AAEL006854: group 4) identified 108 NPC2-like proteins in *An. gambiae s. l.* and in the outgroup species *An. christyi* and *An. epiroticus.* Forty of these were similar to AAEL009557 (AaegML17, group 1) and phylogenetic analysis placed these into six subgroups (blue font in Fig. 9). Thirty proteins were similar to AAEL001650 (AaegML33, group 2) and phylogenetic analysis placed these into five subgroups (gold font in Fig. 9). Nine proteins were similar to AAEL004120 (AaegML1, group 3) and phylogenetic analysis placed these into one group (black font in Fig. 9). Lastly, 29 proteins were similar to AAEL006854 (AaegML13, group 4) and phylogenetic analysis placed these into four subgroups (pink font in Fig. 9). Some groups did not contain a representative from all 8 *Anopheles* species (e.g., subgroup 1a, band e; and subgroups 2a and b), but in general, each of the eight *Anopheles* species had one orthologous copy of each gene in each subgroup. The exceptions to this are in subgroup 1f, group 3 and subgroups 4a and 4b, where gene duplication and further evolution of the gene subgroup is apparent in some species. Indeed, eleven separate *An. gambiae* NPC2 genes are localized in chromosome 2R, nine of which are within region 13E. The phylogeny and gene localizations indicate ancient duplication event(s) within *Anopheles* followed by rapid diversification, and support data suggesting that NPC2 proteins possess a wide-range of lipid and/or sterol binding specificities and diverse functional characteristics, similar to the conclusions suggested by Huang et al. [89] Shi et al. [90], and Jupatanakul et al. [91] given the redundancy of NPC2 genetic structure in *Drosophila* and *Aedes.*

AGAP002851 is the main blood meal-responsive gene (Fig. 9, subgroup 4c, largest font), increasing especially in pre-gravid mosquitoes. Its *Drosophila* orthologue, NPC2e, has so far only been shown to be involved in innate immune signaling [90]. Consequently, the *Anopheles* protein may primarily be responding to the substantial bacterial bloom that occurs in the midgut lumen after blood meal ingestion [95], but it may also have a dual role in sterol homeostasis as has been shown for the *Drosophila* NPC2a protein. It is also important to note that insects cannot synthesize sterol from precursors, but must obtain it from food [96]. The IVM-responsive NPC2 genes (AGAP002848 and AGAP002847) appear to be evolutionarily distinct from the blood meal-responsive NPC2 (group 2 versus group 4, Fig. 9) and were placed in two different subgroups (2a and 2b), suggesting they carry out different functions. The high up-regulation of separate NPC proteins following either a normal blood meal or IVM blood meal paired with the evidence for very diverse binding and function of these proteins in insects suggests direct involvement with the *An. gambiae* response to IVM. Furthermore, the significantly higher upregulation of AGAP002847 in the 6DPE contrast compared to the 2DPE contrast may influence the differential age susceptibility we observed. These data highlight the need for future studies to investigate this potential connection and further characterize these proteins.

### Peritrophic matrix associated genes

The mosquito peritrophic matrix (PM) is a complex structure of chitin-binding and chitin-associated proteins [97, 98]. It lines the luminal side of the midgut and functions to compartmentalize the digestive process in the lumen and protect the midgut epithelium from abrasion during digestion. Our experiment only allows us to follow transcript changes that affect the thick, Type 1 PM in *An. gambiae* females that is secreted upon a blood meal rather than the Type 2 PM constitutively produced in larval guts. The primary Type 1 PM proteins of *An. gambiae* are pre-translated in midgut cell secretory vesicles and released into the lumen upon midgut distension following a blood meal [99, 100], and their transcriptional profiles follow a pattern of high steady-state transcription in NBF and post BF mosquitoes, with only a significant drop at 3 h post BF [64]. Comparison of our dataset with work done by Dinglasan et al. [97] revealed DE of a high number of putative peritrophic matrix-associated genes upon IVM ingestion in both age contrasts (Table 3), suggesting that PM gene transcription is responding directly to IVM treatment. In general the 2DPE contrast showed both an increased number of significantly DE transcripts as well as higher fold-changes than the 6DPE contrast, but interestingly, both age contrasts had DE genes unique to their own age group. Additionally, gene ontology analysis revealed down-regulation of chitin processing transcripts upon blood-feeding (Additional file 9: Figure S3) yet up-regulation of this category after an IVM blood meal (Fig. 6).

**Table 3:** Peritrophic matrix-associated transcripts differentially expressed after IVM ingestion

Gene ID	Description	2IVM:2BF FC	6IVM:6BF FC
AGAP000570	**salivary or midgut protein**	37.57	18.30
AGAP002643	No description	21.93	7.95
AGAP006400	Alkaline phosphatase 2	21.74	5.85
AGAP001819	**Predicted PM protein, Dinglasan et al. 2009** [97] (AgAper25b)	20.03	12.60
AGAP008648	No description	18.65	15.74
AGAP002848	Niemann-Pick Type C-2	16.83	11.92
AGAP007745	No description	15.46	7.79
AGAP006795	Chitin-binding peritrophin (AgAper1)	12.74	6.91
AGAP006432	**ICHIT (AgChitinase)**	9.75	3.13
AGAP006414	chitinase	7.80	4.64
AGAP001199	serine protease	7.12	5.70
AGAP009830	**predicted PM protein, Dinglasan et al. 2009** [97] (AgAper14)	6.84	11.36
AGAP003713	No description	6.23	11.06
AGAP010056	hexosaminidase	4.84	3.32
AGAP006398	No description	3.94	3.38
AGAP006442	No description	2.63	4.35
AGAP004918	fibrinogen	2.51	2.33
AGAP006102	**PRS1 (ribose phosphate diphosphokinase subunit), parital (** ***An. gambiae*** **)**	2.45	2.90
AGAP001193	**No description**	2.26	3.13
AGAP011630	**No description**	2.16	2.28
AGAP008487	**sphingomyelin phosphdiesterase (** ***An. darlingi*** **)**	2.06	5.38
AGAP010733	Vanin-like protein 1	2.05	2.20
AGAP004809	protease m1 zinc metalloprotease	21.09	-
AGAP011302	alkaline phosphatase	12.51	-
AGAP010383	Solute carrier family 15 member 1	9.20	-
AGAP008370	carboxypeptidase A	7.91	-
AGAP011305	alkaline phosphatase	5.32	-
AGAP011453	**transferrin (** ***An. darlingi*** **)**	5.19	-
AGAP004860	protease m1 zinc metalloprotease	4.83	-
AGAP006434	**Predicted PM protein, Dinglasan et al. 2009 [** 97 **] (AgAper57)**	4.46	-
AGAP006433	**Predicted PM protein, Dinglasan et al. 2009 [** 97 **] (AgAper34)**	3.34	-
AGAP008861	female reproductive tract protease GLEANR_896	3.08	-
AGAP006709	Chymotrypsin-1	−4.23	-
AGAP007142	Serine-type enodpeptidase	-	15.35
AGAP005065	**loosely serine-type endopeptidase (** ***Aedes aegypti*** **)**	-	13.64
AGAP010383	Solute carrier family 15 member 1	-	10.06
AGAP006385	**protease or trypsin (late or precurser)**	-	9.29
AGAP008292	Trypsin-4	-	3.47
AGAP008294	Trypsin-3	-	2.94
AGAP006796	peritrophin (Aper9)	-	2.87
AGAP001198	chymotrypsin	-	2.46
AGAP004142	aspartate aminotransferase, cytoplasmic	-	2.18
AGAP006416	Serine protease SP24D	-	2.14
AGAP004883	No description	-	2.04
AGAP008295	Trypsin-2	-	−4.17

Peritrophic matrix associated transcripts significantly differentially expressed following IVM ingestion. Inclusion based on identification of PM proteins by Dinglasan et al. [97], in which the *An. gambiae* adult proteome was thoroughly characterized by mass spectrometry, identifying a total of 209 proteins. Except those indicated as Dinglasan et al., descriptions were provided by VectorBase and descriptions in bold data were annotated based on BLASTp results (*e*-value ≤ 10^−3^) (Additional file 10)

IVM is known to significantly decrease the rate of blood meal digestion [21, 101], even though the glutamate-gated chloride channel target is not expressed in the *An. gambiae* midgut [26]. The significant DE of PM-related transcripts in both age contrasts may be associated with this reduced blood meal processing. There is also good evidence that a primary function of the PM is to bind the abundant heme released from red blood cell digestion, which is toxic and can disrupt cell membranes [102]. The observed increased PM gene transcription may be in response to the prolonged presence of heme in IVM-treated females. Following this hypothesis, it is notable that the less IVM-susceptible age group (2DPE) shows overall higher DE transcription among PM-associated genes in response to IVM than the more IVM-susceptible age group (6DPE), perhaps contributing to their increased tolerance to IVM toxicity.

### Immune response genes

Table 4 lists significantly DE genes previously-linked with mosquito innate immune responses against microorganisms. The older age contrast showed significant DE among many immune-related transcripts. Most were modestly up-regulated, but the expression of two proteases was changed by more than 15-fold (AGAP010968 & AGAP007142). On the other hand, only four immune function genes were significantly DE in the 2DPE contrast.

**Table 4:** Immunity-related transcripts differentially expressed after IVM ingestion

Gene ID	Description	Family	2IVM:2BF FC	6IVM:6BF FC
AGAP010968	Clip-Domain Serine Protease (CLIPA9)	CLIP	5.23	19.69
AGAP007142	Serine-type enodpeptidase	SRP	-	15.35
AGAP009217	Clip-Domain Serine Protease (CLIPB12)	CLIP	-	7.00
AGAP001199	serine protease	SRP	7.12	5.70
AGAP011792	Clip-Domain Serine Protease (CLIPA7)	CLIP	-	2.64
AGAP005552	peptidoglycan recognition protein (Long)	PGRP	-	2.48
AGAP012247	phosphoserine phosphatase	SC	-	2.44
AGAP004918	fibrinogen	FibR	-	2.33
AGAP007386	C-Type Lysozyme	LYS		2.29
AGAP003878	Leucine-rich repeat-transmembrane protein	Anti-parasitic	-	2.21
AGAP006416	Serine protease SP24D	SRP	-	2.14
AGAP003250	Clip-Domain serine protease (CLIPB4)	CLIP	-	2.08
AGAP008645	gambicin anti-microbial peptide	AMP	-	2.03
AGAP004916	Fibrinogen-related	FibR	-	2.02
AGAP009212	Serine Protease Inhibitor (serpin)	SRPN	-	−2.06
AGAP012045	ATP-dependent RNA helixase DDX5/DBP2	Small RNA pathway	3.49	−2.06
AGAP003139	Serine Protease Inhibitor (serpin)	SRPN	-	−2.17
AGAP001979	Class A Scavenger Receptor (SRCR domain) with Serine Protease domain.	SR	-	−3.82
AGAP009509	60S ribosomal protein L9	Small RNA pathway	2.08	-

Transcripts related to immunity significantly differentially expressed transcripts following IVM ingestion. Inclusion and family classifications based on data provided by the insect immunoDB database [108] and other publications. Descriptions in blue text were annotated based on BLASTp results (*e*-value ≤ 10^−3^) (Additional file 10)

*AMP* anti-microbial peptide, *CLIP* clip-domain serine protease, *FibR* fibrinogen-related, *LYSP* lysozyme, *PGRP* peptidoglycan recognition proteins, *SC* signaling-cascade related, *SR* scavenger receptor, *SRP* serine protease, *SRPN* serine protease inhibitor

The up-regulation of so many immune-responsive elements specific to the 6DPE contrast, which have taken a previous blood meal, may suggest that IVM is further influencing the mosquito-microbial interactions and/or innate immune memory from the previous blood meal [103, 104]. It will be important in future studies to examine the impact that IVM may have on the microflora of surviving mosquitoes, as well as how this interaction might influence the previously observed anti-sporogonic effects of the drug [105].

More complex interactions between IVM and the significant DE genes mentioned in all tables above are likely, and some genes have clear linkages across the groups we have highlighted. For example, in the case of immune response genes, it is clear that NPC2 proteins serve as pattern recognition receptors for bacteria lipid moieties. The dramatic up-regulation of some NPC2 transcripts in response to IVM treatment may connect to the up-regulation of downstream immune response pathways (Table 4). Similarly, the PM directly interacts with the microflora bloom that occurs in the gut of blood fed mosquitoes, and Dinglasan et al. [97] previously isolated two NPC2 proteins (AGAP002851, AGAP002848) from dissected *An. gambiae* peritrophic matrices. Also, the PM protein ICHIT (AGAP006432) is immune-responsive following both bacterial and *Plasmodium* invasion [106], but it also has protein-protein aggregation properties, potentially acting as “glue” between chitin-binding and non-chitin binding proteins in the PM [97]. Lastly, detoxification responses connect to cholesterol processing by NPC proteins and to blood digestion and the immune response. It is known that human ABC transporters are essential for sterol homeostasis by efflux of cholesterol and other lipids from cells [107], and anti-oxidant enzymes like the highly up-regulated thioredoxin peroxidase (AGAP011824) are important for redox homeostasis in the gut that may be disrupted in IVM-treated mosquitoes through either prolonged blood meal digestion and/or microflora dysregulation.

## Conclusions

We have shown here that older *An. gambiae* mosquitoes that have ingested previous blood meals are more susceptible to IVM compared to young mosquitoes which have not ingested a prior blood meal. Following from this difference, DE analysis showed that IVM in a blood meal induces significant transcription of more than 100 genes, most of which are up-regulated. Gene expression analyses revealed that IVM in a blood meal mostly induced transcription of non-canonical genes, including PM-associated genes, immune response genes and NPC genes. In regards to the differential susceptibility observed between 2- and 6-day old mosquitoes, several gene expression pattern differences may play a role in producing this phenotype including: a) the NPC gene AGAP002847, which changed from 80.6 upregulated in the 2DPE contrast to 159.5 fold upregulated in the 6DPE contrast, b) the PM-associated genes in which expression was generally higher in the younger, less susceptible age group, suggesting a difference in midgut structure and/or makeup, and c) the immune-related genes, many of which were significantly upregulated in 6DPE mosquitoes only, which suggests an interaction between IVM and immune responses and may be contributing to their increased mortality after IVM ingestion. In contrast, transcriptional evidence for involvement of traditional xenobiotic detoxification mechanisms was limited to the down-regulation of an ABC transporter in 6-day mosquitoes and up-regulation of a GST in 2-day mosquitoes. In addition to new insights into the physiological and molecular effects of IVM on *An. gambiae*, some unexpected findings have added to the knowledge of the under-studied insect NPC proteins and their interesting evolution and diverse functions. While traditional detoxification mechanisms may be party involved, overall the data suggest complex midgut interactions resulting from IVM ingestion that likely involves blood meal digestion physiological responses, midgut microflora, and innate immune responses. These findings have increased our understanding of IVM’s effects on malaria vectors and provide starting points for future studies looking more closely at mosquito-IVM interactions.

### Supporting information

The data set supporting the results of this article is available in the NCBI Sequence Read Archive (SRA) repository. Accession #: SRP060547.

Additionally, expression data from this study is available on the VectorBase Expression Browser under the title “Ivermectin-containing blood meals at different ages” (Seaman et al., 2015) (https://www.vectorbase.org/expression-browser/).

## Additional files

Additional file 1: Figure S1 and Table S1. Average BCV plot values for each replicate sequencing library, plotted against each other (Figure S1) and total number of sequence reads, genes, and reads/gene for each library (Table S1). (TIFF 1079 kb) Additional file 2: Table S2. Primer sequences used for RT-qPCR validation. (XLSX 13 kb) Additional file 3: Table S3.1 – 3.3. Complete list of significantly differentially expressed transcripts in 2DPE groups, including all three contrasts (IVM:BF, BF:NBF, and IVM:NBF). (XLSX 110 kb) Additional file 4: Table S4.1 – 4.3. Complete list of significantly differentially expressed transcripts in 6DPE groups, including all three contrasts (IVM:BF, BF:NBF, and IVM:NBF). (XLSX 76 kb) Additional file 5: Table S5.1 – 5.3. Complete list of significantly differentially expressed transcripts when comparing age groups of the same treatment (6IVM:2IVM, 6BF:2BF, 6NBF:2NBF). (XLSX 65 kb) Additional file 6: Figure S2. Volcano plot and fold-change distribution for the 2DPE:6DPE contrasts. (TIFF 97 kb) Additional file 7: Tables S6.1 – 6.6. Alternative analysis for the identification of IVM-responsive DE transcripts – 2DPE. (XLSX 58 kb) Additional file 8: Tables S7.1 – 7.3. Alternative analysis for the identification of IVM-responsive DE transcripts – 6DPE. (XLSX 40 kb) Additional file 9: Figure S3. Gene Ontology term classifications for DE genes from the BF:NBF contrasts in both age groups. (TIFF 248 kb) Additional file 10: Table S8. BLASTp results for transcripts un-annotated in VectorBase. Queries were run with an e-value threshold of 1x10^−3^. The top 20 hits (lowest e-values) were retained and the most appropriate description was chosen based on these matches. (XLSX 631 kb)

## Abbreviations

ABC: ATP-binding cassette
BCV: Biological coefficient of variation
BF: Blood-fed control
CPM: Counts per million reads
CYP: Cytochrome P450
DE: Differential expression (or differentially expressed)
DMSO: Dimethyl sulfoxide
DPE: Days-post emergence
FC: Fold-change
GST: Glutathione-S-transferase
IRB: Institutional Review Board
IRS: Indoor residual spraying [of insecticides]
IVM: Ivermectin
LLINs: Long-lasting insecticide-treated bed-nets
MDA: Mass drug administration
ML: MD-2 [myeloid differentiation factor-2]-related Lipid-recognition
MOA: Mode of action
NBF: Non-blood-fed
NPC: Niemann-pick type C
PBS: Phosphate buffered saline
PM: Peritrophic matrix
RT-qPCR: Reverse-transcriptase quantitative polymerase chain reaction

## References

[1] Enayati A, Hemingway J (2010). Malaria management: past, present, and future. Annu Rev Entomol.

[2] Corbel V, Guessan RN: Distribution, mechanisms, impact and management of insecticide resistance in malaria vectors : A pragmatic review. In Anopheles mosquitoes - New insights into malaria vectors. First. Edited by Manguin S. Rijeka: InTech; 2013:579–633.

[3] Burg RW, Miller BM, Baker EE, Birnbaum J, Currie SA, Hartman R, Kong Y-L, Monaghan RL, Olson G, Putter I, Tunac JB, Wallick H, Stapley EO, Oiwa R, Omura S (1979). Avermectins, new family of potent anthelmintic agents: producing organism and fermentation. Antimicrob Agents Chemother.

[4] Campbell W (1991). Ivermectin as an antiparasitic agent for use in humans. Annu Rev Microbiol.

[5] Hotez PJ (2007). Control of onchocerciasis — the next generation. Lancet.

[6] Sheriff JC, Kotze AC, Sangster NC, Hennessy DR (2005). Effect of ivermectin on feeding by *Haemonchus contortus* in vivo. Vet Parasitol.

[7] Venco L, McCall JW, Guerrero J, Genchi C (2004). Efficacy of long-term monthly administration of ivermectin on the progress of naturally acquired heartworm infections in dogs. Vet Parasitol.

[8] Meinking TL, Taplin D, Hermida JL, Pardo R, Kerdel FA (1995). The treatment of scabies with ivermectin. N Engl J Med.

[9] Cramer LG, Carvalho LA, Bridi AA, Amaral NK, Barrick RA (1988). Efficacy of topically applied ivermectin against *Boophilus microplus* (Canestrini, 1887) in cattle. Vet Parasitol.

[10] Rugg D, Gogolewski RP, Barrick RA, Eagleson JS (1997). Efficacy of ivermectin controlled-release capsules for the control and prevention of nasal bot infestations in sheep. Aust Vet J.

[11] Guzzo CA, Furtek CI, Porras AG, Chen C, Tipping R, Clineshmidt CM, Sciberras DG, Hsieh JY-K, Lasseter KC (2002). Safety, tolerability, and pharmacokinetics of escalating high doses of ivermectin in healthy adult subjects. J Clin Pharmacol.

[12] De Sole G, Remme J, Awadzi K, Accorsi S, Alley ES, Ba O, Dadzie KY, Giese J, Karam M, Keita FM (1989). Adverse reactions after large-scale treatment of onchocerciasis with ivermectin: combined results from eight community trials. Bull World Health Organ.

[13] Twum-Danso NA (2003). Serious adverse events following treatment with ivermectin for onchocerciasis control: a review of reported cases. Filaria J.

[14] Mascari TM, Foil LD (2010). Oral treatment of rodents with ivermectin for the control of *Phlebotomus papatasi* (Diptera: Psychodidae) under laboratory conditions. Vet Parasitol.

[15] Pooda SH, Mouline K, De Meeûs T, Bengaly Z, Solano P (2013). Decrease in survival and fecundity of *Glossina palpalis gambiensis* vanderplank 1949 (*Diptera: Glossinidae*) fed on cattle treated with single doses of ivermectin. Parasit Vectors.

[16] Chaccour CJ, Kobylinski KC, Bassat Q, Bousema T, Drakeley C, Alonso P, Foy BD (2013). Ivermectin to reduce malaria transmission: a research agenda for a promising new tool for elimination. Malar J.

[17] Bockarie MJ, Hii JLK, Alexander NDE, Bockarie F, Dagoro H, Kazura JW, Alpers MP (1999). Mass treatment with ivermectin for filariasis control in Papua New Guinea: Impact on mosquito survival. Med Vet Entomol.

[18] Butters MP, Kobylinski KC, Deus KM, da Silva IM, Gray M, Sylla M, Foy BD (2012). Comparative evaluation of systemic drugs for their effects against *Anopheles gambiae*. Acta Trop.

[19] Chaccour C, Lines J, Whitty CJM (2010). Effect of ivermectin on *Anopheles gambiae* mosquitoes fed on humans: The potential of oral insecticides in malaria control. J Infect Dis.

[20] Foley DH, Bryan JH, Lawrence GW (2000). The potential of ivermectin to control the malaria vector *Anopheles farauti*. Trans R Soc Trop Med Hyg.

[21] Kobylinski KC, Deus KM, Butters MP, Hongyu T, Gray M, da Silva IM, Sylla M, Foy BD (2010). The effect of oral anthelmintics on the survivorship and re-feeding frequency of anthropophilic mosquito disease vectors. Acta Trop.

[22] Sylla M, Kobylinski KC, Gray M, Chapman PL, Sarr MD, Rasgon JL, Foy BD (2010). Mass drug administration of ivermectin in south-eastern Senegal reduces the survivorship of wild-caught, blood fed malaria vectors. Malar J.

[23] Tesh RB, Guzman H (1990). Mortality and infertility in adult mosquitoes after the ingestion of blood containing ivermectin. Am J Trop Med Hyg.

[24] Alout H, Krajacich BJ, Meyers JI, Grubaugh ND, Brackney DE, Kobylinski KC, Diclaro JW, Bolay FK, Fakoli LS, Diabaté A, Dabiré RK, Bougma RW, Foy BD (2014). Evaluation of ivermectin mass drug administration for malaria transmission control across different West African environments. Malar J.

[25] Kobylinski KC, Sylla M, Chapman PL, Sarr MD, Foy BD (2011). Ivermectin mass drug administration to humans disrupts malaria parasite transmission in Senegalese villages. Am J Trop Med Hyg.

[26] Meyers JI, Gray M, Kuklinski W, Johnson LB, Snow CD, Black WC, Partin KM, Foy BD (2015). Characterization of the target of ivermectin, the glutamate-gated chloride channel, from *Anopheles gambiae*. J Exp Biol.

[27] David J-P, Coissac E, Melodelima C, Poupardin R, Riaz MA, Chandor-Proust A, Reynaud S (2010). Transcriptome response to pollutants and insecticides in the dengue vector *Aedes aegypti* using next-generation sequencing technology. BMC Genomics.

[28] Vontas J, Blass C, Koutsos AC, David J-P, Kafatos FC, Louis C, Hemingway J, Christophides GK, Ranson H (2005). Gene expression in insecticide resistant and susceptible *Anopheles gambiae* strains constitutively or after insecticide exposure. Insect Mol Biol.

[29] Yoon KS, Strycharz JP, Baek JH, Sun W, Kim JH, Kang JS, Pittendrigh BR, Lee SH, Clark JM (2011). Brief exposures of human body lice to sublethal amounts of ivermectin over-transcribes detoxification genes involved in tolerance. Insect Mol Biol.

[30] Pohl PC, Klafke GM, Carvalho DD, Martins JR, Daffre S, da Silva Vaz I, Masuda A (2011). ABC transporter efflux pumps: a defense mechanism against ivermectin in *Rhipicephalus (Boophilus) microplus*. Int J Parasitol.

[31] Kane NS, Hirschberg B, Qian S, Hunt D, Thomas B, Brochu R, Ludmerer SW, Zheng Y, Smith M, Arena JP, Cohen CJ, Schmatz D, Warmke J, Cully DF (2000). Drug-resistant *Drosophila* indicate glutamate-gated chloride channels are targets for the antiparasitics nodulisporic acid and ivermectin. Proc Natl Acad Sci U S A.

[32] Luo L, Sun Y-J, Wu Y-J (2013). Abamectin resistance in *Drosophila* is related to increased expression of P-glycoprotein via the dEGFR and dAkt pathways. Insect Biochem Mol Biol.

[33] Wu TD, Nacu S (2010). Fast and SNP-tolerant detection of complex variants and splicing in short reads. Bioinformatics.

[34] Wu TD, Watanabe CK (2005). GMAP: a genomic mapping and alignment program for mRNA and EST sequences. Bioinformatics.

[35] Bolger AM, Lohse M, Usadel B (2014). Trimmomatic: a flexible trimmer for Illumina sequence data. Bioinformatics.

[36] Giraldo-Calderon GI, Emrich SJ, MacCallum RM, Maslen G, Dialynas E, Topalis P, Ho N, Gesing S, Madey G, Collins FH, Lawson D (2015). VectorBase: an updated bioinformatics resource for invertebrate vectors and other organisms related with human diseases. Nucleic Acids Res.

[37] Robinson MD, McCarthy DJ, Smyth GK (2010). edgeR: a Bioconductor package for differential expression analysis of digital gene expression data. Bioinformatics.

[38] R Core Team: R: A language and environment for statistical computing. *R Foundation for Statistical Computing* 2014:http://www.R-project.org/.

[39] Benjamini Y, Hochberg Y (1995). Controlling the false discovery rate: a practical and powerful approach to multiple testing. J R Stat Soc Ser B.

[40] Reimand J, Arak T, Vilo J (2011). g:Profiler--a web server for functional interpretation of gene lists (2011 update). Nucleic Acids Res.

[41] Felsenstein J (1989). PHYLIP - Phylogeny Inference Package (Version 3.2). Cladistics.

[42] Kosiol C, Goldman N (2005). Different versions of the dayhoff rate matrix. Mol Biol Evol.

[43] Dayhoff MO, Schwartz RM, Orcutt BC, Dayhoff M (1978). A model of evolutionary change in proteins. Atlas of Protein Sequence and Structure.

[44] Saitou N, Nei M (1987). The neighbor-joining method: a new method for reconstructing phylogenetic trees. Mol Biol Evol.

[45] Stöver BC, Müller KF (2010). TreeGraph 2: combining and visualizing evidence from different phylogenetic analyses. BMC Bioinformatics.

[46] PrimerQuest [http://www.idtdna.com/primerquest/home/index ] Accessed Oct 3, 2014.

[47] Wilkins EE, Howell PI, Benedict MQ (2006). IMP PCR primers detect single nucleotide polymorphisms for Anopheles gambiae species identification, Mopti and Savanna rDNA types, and resistance to dieldrin in *Anopheles arabiensis*. Malar J.

[48] Livak KJ, Schmittgen TD (2001). Analysis of relative gene expression data using real-time quantitative PCR and the 2(−delta delta C(T)) method. Methods.

[49] Styer LM, Carey JR, Wang J-L, Scott TW (2007). Mosquitoes do senesce: Departure from the paradigm of constant mortality. Am J Trop Med Hyg.

[50] Rajatileka S, Burhani J, Ranson H (2011). Mosquito age and susceptibility to insecticides. Trans R Soc Trop Med Hyg.

[51] Jones CM, Sanou A, Guelbeogo WM, Sagnon N, Johnson PCD, Ranson H (2012). Aging partially restores the efficacy of malaria vector control in insecticide-resistant populations of *Anopheles gambiae* s.l. from Burkina Faso. Malar J.

[52] Chouaibou MS, Chabi J, Bingham GV, Knox TB, N’Dri L, Kesse NB, Bonfoh B, Jamet HVP (2012). Increase in susceptibility to insecticides with aging of wild *Anopheles gambiae* mosquitoes from Côte d’Ivoire. BMC Infect Dis.

[53] Glunt KD, Thomas MB, Read AF (2011). The effects of age, exposure history and malaria infection on the susceptibility of *Anopheles* mosquitoes to low concentrations of pyrethroid. PLoS One.

[54] Oliver SV, Brooke BD (2014). The effect of multiple blood-feeding on the longevity and insecticide resistant phenotype in the major malaria vector *Anopheles arabiensis* (Diptera: Culicidae). Parasit Vectors.

[55] Halliday WR, Feyereisen R (1987). Why does DDT toxicity change after a blood meal in adult female *Culex pipiens*?. Pestic Biochem Physiol.

[56] Spillings BL, Coetzee M, Koekemoer LL, Brooke BD (2008). The effect of a single blood meal on the phenotypic expression of insecticide resistance in the major malaria vector *Anopheles funestus*. Malar J.

[57] Clements AN (1992). The Biology of Mosquitoes, Vol. 1: Development Structure and Reproduction.

[58] Beyenbach KW (2003). Transport mechanisms of diuresis in Malpighian tubules of insects. J Exp Biol.

[59] Hibbs RE, Gouaux E (2011). Principles of activation and permeation in an anion-selective Cys-loop receptor. Nature.

[60] Campbell WC, Fisher MH, Stapley EO, Albers-Schönberg G, Jacob TA (1983). Ivermectin: a potent new antiparasitic agent. Science.

[61] Marinotti O, Nguyen Q, Calvo E, James AA, Ribeiro JMC (2005). Microarray analysis of genes showing variable expression following a blood meal in *Anopheles gambiae*. Insect Mol Biol.

[62] Vannini L, Augustine Dunn W, Reed TW, Willis JH (2014). Changes in transcript abundance for cuticular proteins and other genes three hours after a blood meal in *Anopheles gambiae*. Insect Biochem Mol Biol.

[63] Dana AN, Hong YS, Kern MK, Hillenmeyer ME, Harker BW, Lobo NF, Hogan JR, Romans P, Collins FH (2005). Gene expression patterns associated with blood-feeding in the malaria mosquito *Anopheles gambiae*. BMC Genomics.

[64] Marinotti O, Calvo E, Nguyen QK, Dissanayake S, Ribeiro JMC, James AA (2006). Genome-wide analysis of gene expression in adult *Anopheles gambiae*. Insect Mol Biol.

[65] Scott TW, Takken W (2012). Feeding strategies of anthropophilic mosquitoes result in increased risk of pathogen transmission. Trends Parasitol.

[66] Enayati AA, Ranson H, Hemingway J (2005). Insect glutathione transferases and insecticide resistance. Insect Mol Biol.

[67] Tang AH, Tu C-PD (1994). Biochemical characterization of *Drosophila* glutathione S-transferases D1 and D21. J Biol Chem.

[68] Vontas JG, Small GJ, Hemingway J (2001). Glutathione S-transferases as antioxidant defence agents confer pyrethroid resistance in *Nilaparvata lugens*. Biochem J.

[69] Wang JY, McCommas S, Syvanen M (1991). Molecular-cloning of a glutathione S-transferase overproduced in an insecticide-resistant strain of the housefly (*Musca domestica*). Mol Gen Genet.

[70] Bariami V, Jones CM, Poupardin R, Vontas J, Ranson H (2012). Gene amplification, ABC transporters and cytochrome P450s: unraveling the molecular basis of pyrethroid resistance in the dengue vector, *Aedes aegypti*. PLoS Negl Trop Dis.

[71] David J, Ismail HM, Chandor-proust A, Paine IMJ (2013). Role of cytochrome P450s in insecticide resistance: impact on the control of mosquito-borne diseases and use of insecticides on Earth. Philos Trans R Soc B.

[72] Félix R, Silveira H, Khan F (2010). The role of *Anopheles gambiae* P450 Cytochrome in insecticide resistance and infection. Insecticides - Pest Engineering.

[73] Scott JG (1999). Cytochromes P450 and insecticide resistance. Insect Biochem Mol Biol.

[74] Lanning CL, Fine RL, Corcoran JJ, Ayad HM, Rose RL, Abou-Donia MB (1996). Tobacco budworm P-glycoprotein: biochemical characterization and its involvement in pesticide resistance. Biochim Biophys Acta.

[75] Jones CM, Toé HK, Sanou A, Namountougou M, Hughes A, Diabaté A, Dabiré R, Simard F, Ranson H (2012). Additional selection for insecticide resistance in urban malaria vectors: DDT resistance in *Anopheles arabiensis* from Bobo-Dioulasso. Burkina Faso. PLoS One.

[76] Pohl PC, Klafke GM, Júnior JR, Martins JR, da Silva VI, Masuda A (2012). ABC transporters as a multidrug detoxification mechanism in *Rhipicephalus (Boophilus) microplus*. Parasitol Res.

[77] Matowo J, Jones CM, Kabula B, Ranson H, Steen K, Mosha F, Rowland M, Weetman D (2014). Genetic basis of pyrethroid resistance in a population of *Anopheles arabiensis*, the primary malaria vector in Lower Moshi, north-eastern Tanzania. Parasit Vectors.

[78] Buss DS, McCaffery AR, Callaghan A (2002). Evidence for p-glycoprotein modification of insecticide toxicity in mosquitoes of the *Culex pipiens* complex. Med Vet Entomol.

[79] James CE, Davey MW (2009). Increased expression of ABC transport proteins is associated with ivermectin resistance in the model nematode *Caenorhabditis elegans*. Int J Parasitol.

[80] Podsiadlowski L, Matha V, Vilcinskas A (1998). Detection of a P-glycoprotein related pump in *Chironomus* larvae and its inhibition by verapamil and cyclosporin A. Comp Biochem Physiol Part B.

[81] Liscum L, Sturley SL (2004). Intracellular trafficking of Niemann-Pick C proteins 1 and 2: obligate components of subcellular lipid transport. Biochim Biophys Acta.

[82] Chang T-Y, Chang CCY, Ohgami N, Yamauchi Y (2006). Cholesterol sensing, trafficking, and esterification. Annu Rev Cell Dev Biol.

[83] Vanier MT (2010). Niemann-Pick disease type C. Orphanet J Rare Dis.

[84] Willenborg M, Schmidt CK, Braun P, Landgrebe J, von Figura K, Saftig P, Eskelinen E-L (2005). Mannose 6-phosphate receptors, Niemann-Pick C2 protein, and lysosomal cholesterol accumulation. J Lipid Res.

[85] Ko DC, Binkley J, Sidow A, Scott MP (2003). The integrity of a cholesterol-binding pocket in Niemann-Pick C2 protein is necessary to control lysosome cholesterol levels. Proc Natl Acad Sci U S A.

[86] Shimazu R, Akashi S, Ogata H, Nagai Y, Fukudome K, Miyake K, Kimoto M (1999). MD-2, a molecule that confers lipopolysaccharide responsiveness on Toll-like receptor 4. J Exp Med.

[87] Viriyakosol S, Tobias PS, Kitchens RL, Kirkland TN (2001). MD-2 Binds to Bacterial Lipopolysaccharide. J Biol Chem.

[88] Inohara N, Nuez G (2002). ML - a conserved domain involved in innate immunity and lipid metabolism. Trends Biochem Sci.

[89] Huang X, Warren JT, Buchanan J, Gilbert LI, Scott MP (2007). *Drosophila* Niemann-Pick type C-2 genes control sterol homeostasis and steroid biosynthesis: a model of human neurodegenerative disease. Development.

[90] Shi XZ, Zhong X, Yu XQ (2012). *Drosophila melanogaster* NPC2 proteins bind bacterial cell wall components and may function in immune signal pathways. Insect Biochem Mol Biol.

[91] Jupatanakul N, Sim S, Dimopoulos G (2014). *Aedes aegypti* ML and Niemann-Pick type C family members are agonists of dengue virus infection. Dev Comp Immunol.

[92] Dong Y, Aguilar R, Xi Z, Warr E, Mongin E, Dimopoulos G (2006). *Anopheles gambiae* immune responses to human and rodent *Plasmodium* parasite species. PLoS Pathog.

[93] Huang X, Suyama K, Buchanan J, Zhu AJ, Scott MP (2005). A *Drosophila* model of the Niemann-Pick type C lysosome storage disease: dnpc1a is required for molting and sterol homeostasis. Development.

[94] Fluegel ML, Parker TJ, Pallanck LJ (2006). Mutations of a *Drosophila* NPC1 gene confer sterol and ecdysone metabolic defects. Genetics.

[95] Pumpuni CB, Demaio J, Kent M, Davis JR, Beier JC (1996). Bacterial population dynamics in three Anopheline species: the impact on *Plasmodium* sporogonic development. Am J Trop Med Hyg.

[96] Clark AJ, Block K (1959). The absence of sterol synthesis in insects. J Biol Chem.

[97] Dinglasan RR, Devenport M, Florens L, Johnson JR, McHugh CA, Donnelly-Doman M, Carucci DJ, Yates JR, Jacobs-Lorena M (2009). The *Anopheles gambiae* adult midgut peritrophic matrix proteome. Insect Biochem Mol Biol.

[98] Shao L, Devenport M, Jacobs-Lorena M (2001). The peritrophic matrix of hematophagous insects. Arch Insect Biochem Physiol.

[99] Devenport M, Fujioka H, Jacobs-Lorena M (2004). Storage and secretion of the peritrophic matrix protein Ag-Aper1 and trypsin in the midgut of *Anopheles gambiae*. Insect Mol Biol.

[100] Devenport M, Fujioka H, Donnelly-Doman M, Shen Z, Jacobs-Lorena M (2005). Storage and secretion of Ag-Aper14, a novel peritrophic matrix protein, and Ag-Muc1 from the mosquito *Anopheles gambiae*. Cell Tissue Res.

[101] Fritz ML, Siegert PY, Walker ED, Bayoh MN, Vulule JR, Miller JR (2009). Toxicity of bloodmeals from ivermectin-treated cattle to Anopheles gambiae s. l. Ann Trop Med Parasitol.

[102] Pascoa V, Oliveira PL, Dansa-Petretski M, Silva JR, Alvarenga PH, Jacobs-Lorena M, Lemos FJA (2002). *Aedes aegypti* peritrophic matrix and its interaction with heme during blood digestion. Insect Biochem Mol Biol.

[103] Ziauddin J, Schneider DS (2012). Where does innate immunity stop and adaptive immunity begin?. Cell Host Microbe.

[104] Rodrigues J, Brayner FA, Alves LC, Dixit R, Barillas-Mury C (2010). Hemocyte differentiation mediates innante immune memory in *Anopheles gambiae* mosquiteos. Science.

[105] Kobylinski KC, Foy BD, Richardson JH (2012). Ivermectin inhibits the sporogony of *Plasmodium falciparum* in *Anopheles gambiae*. Malar J.

[106] Dimopoulos G, Seeley D, Wolf A, Kafatos FC (1998). Malaria infection of the mosquito *Anopheles gambiae* activates immune-responsive genes during critical transition stages of the parasite life cycle. EMBO J.

[107] Yu L, Hammer RE, Li-Hawkins J, Von Bergmann K, Lutjohann D, Cohen JC, Hobbs HH (2002). Disruption of Abcg5 and Abcg8 in mice reveals their crucial role in biliary cholesterol secretion. Proc Natl Acad Sci U S A.

[108] Waterhouse RM, Kriventseva EV, Meister S, Xi Z, Alvarez KS, Bartholomay LC, Barillas-Mury C, Bian G, Blandin S, Christensen BM, Dong Y, Jiang H, Kanost MR, Koutsos AC, Levashina EA, Li J, Ligoxygakis P, Maccallum RM, Mayhew GF, Mendes A, Michel K, Osta MA, Paskewitz S, Shin SW, Vlachou D, Wang L, Wei W, Zheng L, Zou Z, Severson DW (2007). Evolutionary dynamics of immune-related genes and pathways in disease-vector mosquitoes. Science.

