# Supplementary figures and images for: Age and prior blood feeding of Anopheles gambiae influences their susceptibility and gene expression patterns to ivermectin-containing blood meals
Source: BMC Genomics. 2015 Oct 15;16:797. doi: 10.1186/s12864-015-2029-8 (PMC4608139; doi:10.1186/s12864-015-2029-8)

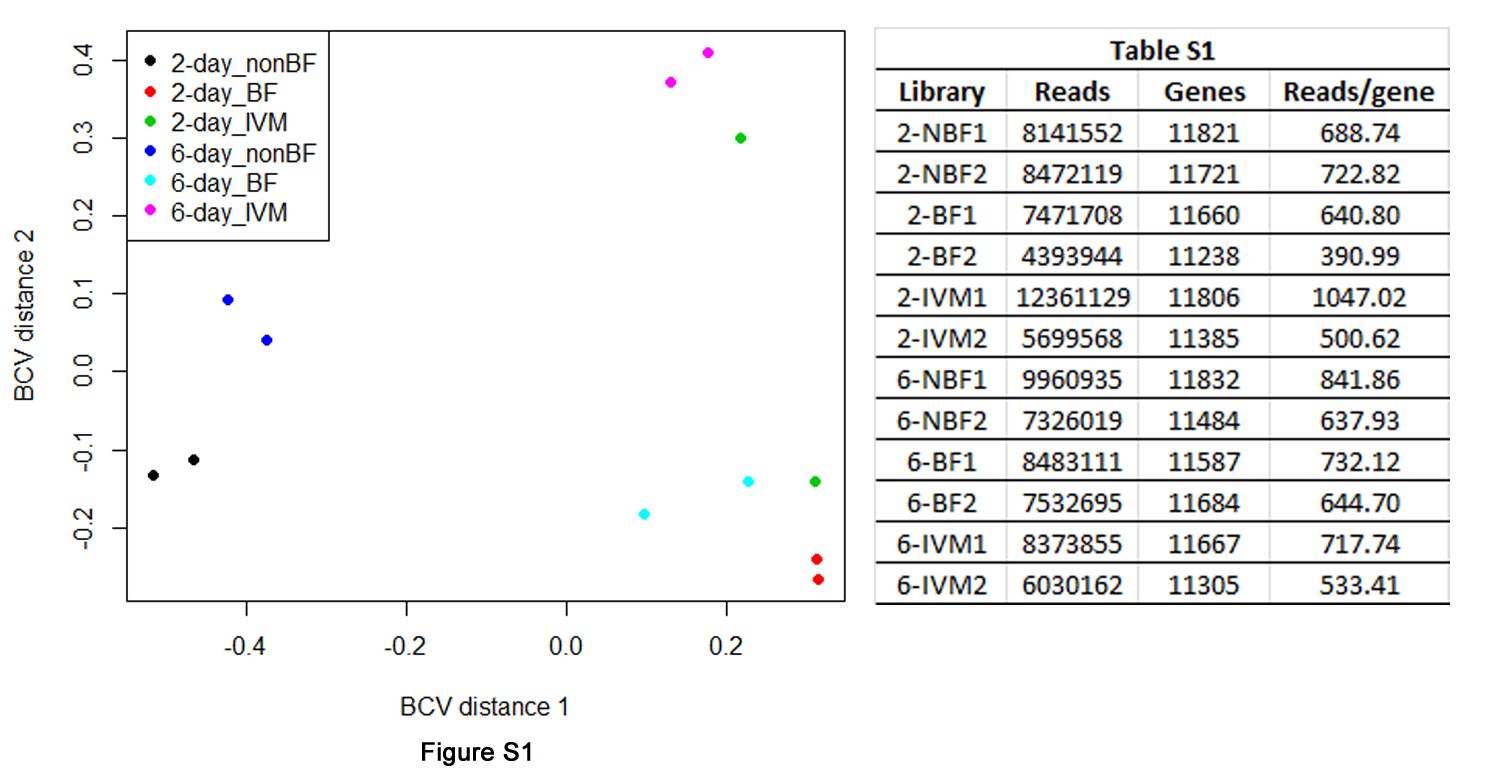

Supplement: Additional file 1: Figure S1 and Table S1. — Average BCV plot values for each replicate sequencing library, plotted against each other (Figure S1) and total number of sequence reads, genes, and reads/gene for each library (Table S1). (TIFF 1079 kb) [file 12864_2015_2029_MOESM1_ESM.tif]
